# MUC1 as a target for CAR‐T therapy in head and neck squamous cell carinoma

**DOI:** 10.1002/cam4.2733

**Published:** 2019-12-04

**Authors:** Zi Mei, Kai Zhang, Alfred King‐Yin Lam, Junwen Huang, Feng Qiu, Bin Qiao, Yi Zhang

**Affiliations:** ^1^ Department of Oral and Maxillofacial Surgery The First Affiliated Hospital of Zhengzhou University Zhengzhou China; ^2^ Biotherapy Center The First Affiliated Hospital of Zhengzhou University Zhengzhou Henan China; ^3^ Cancer Molecular Pathology and Griffith Medical School Griffith University Gold Coast Queensland Australia

**Keywords:** CAR‐T, head and neck squamous cell carcinoma, IL22, MUC1

## Abstract

The modification of chimeric antigen receptor (CAR) endowing T cells with tumor‐specific cytotoxicity induces antitumor immunity. However, the structural characteristics of solid tumors, the loss of specific antigens, and the strong immunosuppressive environment are challenges to treat solid tumors with CAR‐T therapy. The purpose of our study was to find and verify the potentials of CAR‐T therapies for patients with head and neck squamous cell carcinoma (HNSCC). First, we selected MUC1 as our research target and verified its differential expression in cancer tissues and adjacent non‐neoplastic tissues (ANNT). Next, we constructed a second‐generation CAR and validated the cytotoxic function in vitro. In our study, we found that exogenous addition human IL22 recombinant protein could increase the MUC1 expression and enhance the function of T cells. In addition, we constructed a fourth‐generation CAR that secretes IL22. Finally, we verified the antitumor function of two different CAR‐T cells in vitro and in vivo, respectively. CAR‐MUC1‐IL22 T cells were found to have a stronger and more effective cytotoxic function against MUC1 + HNSCC cells. Taken together, these results demonstrate the potential effectiveness of CAR‐T in the treatment of patients with HNSCC and provide evidence‐based of MUC1 + CAR‐T therapy.

## Graphical abstract

Identified the expression of MUC1 is higher in head and neck squamous cell carcinomas than non‐neoplastic tissues. Construct the second‐generation CAR‐T cell (CAR‐MUC1 T cell) and the fourth‐generation CAR‐T cell (CAR‐MUC1‐IL22 T cell) structure, respectively, and verify the function of different T cells to target tumors in vitro and in vivo. Since IL22 can upregulate the expression of MUC1, the fourth‐generation CAR‐T can kill HNSCC cells more effectively.

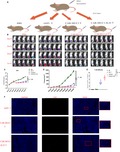

## 1 INTRODUCTION

Head and neck carcinoma is the sixth‐highest incidence cancer in the world, among them, squamous cell carcinoma is the major pathological type.^1^ With the improvement of current treatments, the prognosis of head and neck squamous cell carcinoma remains unsatisfactory.^2^ Clinical treatments of HNSCC mainly include surgery, radiation therapy, and chemotherapy. Surgery and chemotherapy are commonly used for patients with cancer recurrence and metastasis, but the prognosis remains unfavorable. Cetuximab is the first Food and Drug Administration (FDA)‐approved targeted drug for HNSCC which is a monoclonal antibody (mAb) targeting epidermal growth factor receptor (EGFR). The addition of cetuximab to platinum‐based chemotherapy (such as cisplatin) slightly increased the survival rate of patients by 2.7 months, reducing the relative risk of death by 20%.^3^ Patients with HNSCC having recurrence or metastases treated with cisplatin alone had a worse prognosis with less than 5% surviving for a year. Therefore, new treatments that could improve the outcomes of patients with head and neck squamous cell carcinoma are needed.

Human leukocyte antigen (HLA) complex expresses treated tumor antigen peptide to T lymphocytes. Tumor cells can reduce the recognition of CD8^+^ cells by altering HLA class I expression. HNSCC cells use a variety of mechanisms to achieve immune evasion and avoid complete loss of HLA expression.^4^ In the recent years, adoptive cell therapy (ACT) has achieved significant success in the treatment of hematological malignancies.^5^ More especially, the ACT of T cells expressing a chimeric antigen receptor has received much attention. There are four parts in structure of CARs: an extracellular antigen recognition region with single‐chain antibody fragment (scFvs), which derive from a monoclonal antibody and recognize specific tumor‐related antigens without major histocompatibility complex (MHC); a transmembrane domain that is usually derived from CD8, CD3‐ζ, CD4, OX40; an intracellular signaling domain including only one activating domain (usually a portion of the ζ chain in the TCR complex)or/and a costimulatory receptor (CD28, CD27, 4‐1BB, or OX40) and an extracellular hinge domain.^6^ Thus, CAR‐T cells function in a non‐MHC‐dependent restricted manner. By introducing CAR structure into T cells, T cells may be activated and recognized by the tumor‐associated antigens (TAA), release perforin, granulemycin, and a variety of cytokines to exert powerful antitumor function.^7^

CAR‐CD19 T cells have shown exciting results in the treatment of pediatric hematologic malignancies.^8^ Due to the histological structural characteristics of solid tumors, the absence of specific antigens, clinical results for solid tumors have not been remarkable. Therefore, it is very important to choose an effective and reliable antigen to treat solid tumors.

MUC1 belongs to the mucin family and is often expressed on varieties of epithelial cell types. It is often overexpressed during tumor transformation, possibly as a result of glycosylation changes. Mucin plays a central role in maintaining homeostasis and promoting cell survival.^9^ The expression of MUC1 is not limited to the cell membrane, but also expressed in the cytoplasm.^10^ These results make easier to distinguish between tumor cells and non‐neoplastic cells, making targeted therapies more precise.^11^

MUC1‐targeting CAR‐T cells have been successfully used in the treatment of non‐small‐cell lung cancer, breast cancer^12^ and in clinic trials for patients with advanced refractory solid tumors (ClinicalTrials.gov Identifier: NCT02587689). Moreover, MUC1 is higher expressed in HNSCC tissues compared to adjacent non‐neoplastic tissues.^13^ In this study, we constructed a second‐generation CAR targeting MUC1 and tested the cytotoxic function against HNSCC cell lines in vitro.

IL22 is a cytokine belonging to the IL10 family and is preferentially expressed in the subset of Th17 cells.^14^ IL22 is produced by CD4 T cells at high levels, and by NK cells at low levels.^15^ Increasing of this cytokines can be seen in some immunosuppressive diseases such as interstitial lung disease, etc^16^ Reportedly, exogenously added human IL22 recombinant protein can increase the expression of MUC1 in tumor's inflammatory microenvironment.^17^

HNSCC is an immunosuppressive disease and has inflammatory tumor microenvironment. 18 Therefore, in this study, we also linked the IL22 to CAR‐T therapy and constructed a fourth‐generation CAR structure that could target MUC1 and secrete IL22 cytokine. Our aim is to verify that its killing function is stronger than second‐generation CAR in vitro and in vivo.

## 2 MATERIALS AND METHODS

### 2.1 Cell lines and culture

Human tongue squamous carcinoma cell lines (Cal27, SCC15, SCC25) were purchased from American Tissue Culture Collection (ATCC) and human tongue squamous carcinoma cell line (Cal33) and human oropharyngeal squamous carcinoma cell line (HN4) was obtained from Shanghai Ninth People's Hospital, Shanghai Jiaotong University School of Medicine. They were all cultured in Dulbecco's Modified Eagle Medium (DMEM‐F12) complete medium composes of 10% fetal bovine serum (FBS, HyClone) and 100 U/mL penicillin, 100 μg/mL streptomycin (Invitrogen). The Luciferase‐green fluorescent protein (GFP)‐expressing cell line HN4 was obtained by transfecting the tumor cells with the supernatant containing Luciferase‐GFP virus, and the GFP channel was sorted by FACS Aria^TM^ cell sorter (BD Biosciences). Luciferase‐GFP was expressed stably and cultured in DMEM‐F12 complete medium and mainly used in animal experiments. *hTERT* ^+^–oral mucosal epithelial cell line (OME) is an immortalized cell line. It was formed from *hTERT* ^+^ transferred into OME cells.^19^ These cells were cultured in DMEM‐F12 complete medium.

### 2.2 Preparation and culture of CD3^+^ T cells

Human peripheral blood mononuclear cells (PBMCs) were isolated by lymphocyte stratification (Ficoll) density gradient centrifugation. CD3^+^ T cells were magnetically sorted using CD3 magnetic beads. T cells were activated with CD3/CD28 antibody (1 µL/ 10^7^ cells) two days prior to use. CD3^+^ T cells were cultured with Roswell Park Memorial Institute (RPMI) 1640 medium with the addition of 100 IU/mL human recombinant interleukin 2 (IL‐2) (PeproTech) and 2 mmol/L L‐glutamine (Gibco/Life Technologies/Thermo Fisher Scientific).

### 2.3 Primary tumor samples

The tissues collected included 52 paired HNSCC tumor tissues (typically 2 cm^3^) and adjacent non‐neoplastic tissues (distance from the tumor tissue is more than 3‐5 cm) which were freshly obtained from the Department of Oral and Maxillofacial Surgery of the First Affiliated Hospital of Zhengzhou University (Zhengzhou, China) between 2017 and 2019. All these patients had not received any prior radiotherapy, chemotherapy, or any other therapy. These tissues were washed with phosphate‐buffered saline (PBS) and cut into small pieces. TRIzol (Invitrogen) was added to a small part of these tissues in preparation for RNA extraction according to the standard protocol.

### 2.4 Immunofluorescence (IF)

Fresh tumor tissues of mice were obtained and embedded in paraffin. CD3 immunofluorescence antibody (BV421, mouse anti‐human, Becton, Dickinson) was incubated overnight at 4°C. On the following day, after 1 hour of room temperature recovery, the tissue samples were incubated with anti‐mouse secondary antibodies for 1 hour, followed by 4′, 6‐diamidino‐2‐phenylindole (DAPI) nuclear counterstaining. Finally, fluorescent quencher was used to seal the slide, and the tissues were stored in a dry box. Tissue samples were photographed under the microscope within 5 days.

### 2.5 CAR‐MUC1 construct synthesis

The second‐generation CAR structure is composed of the MUC1 mAb scFv (VH: HMFG2, VL: SM3) sequence linked to the CD8α hinge and transmembrane region, the intracellular domain of 4‐1BB and CD3ζ signal peptide. These structures were synthesized and cloned onto the pCDH‐GFP lentiviral vector by PPL (Public Protein/Plasmid Library, China). The fourth‐generation structure was based on the second generation structure and the CDS region of IL22 was connected by 2A peptide. The final sequence is “Lenti‐2G‐Muc1‐CAR‐T‐2A‐IL22” plasmid.

### 2.6 Retrovirus production and T‐cell transduction

Lentiviral granule was made of the primary and packaging plasmids (psPAX2 and pMD.2G) transfected into human embryonic kidney HEK 293T cells (Sigma‐Aldrich). On the first day, 293T cells were evenly seeded in the 6‐well plate with 1640 medium. On the second day, the RPMI‐1640 medium was discarded from the 293T cells and replaced with Opti‐MEM (Gibco/Thermo Fisher Scientific), with an additional 1.5 µg primary plasmid, 1 µg pMD.2G, 1.5 µg psPAX2 and 8 µL lipofectamine 3000 (Invitrogen) in each well. After 14‐18 hours, the RPMI‐1640 medium was replaced, and the supernatant was collected 48 hours later. The viral supernatant collected was concentrated 10‐20 times with virus concentration reagent, and the concentrated viral granules were stored at −8°C.

Two days postactivation, CD3^+^ T cells were evenly distributed into 24‐well plates at 10^6^ cells per well. The concentrated viral granules were added to the 24‐well plate and 8 µg/mL polybrene (Sigma‐Aldrich). After 24 hours, the CD3^+^ T cells were returned to the RPMI‐1640 medium at half the original volume.

### 2.7 T‐cell function assay and Flow cytometry analysis

T cells were coincubated with target cells at an effect: target cell ratio (E/T ratio) at different ratios, three subwells were set at a time and coincubated in a 96‐well plate. Next, supernatants and cells were collected after coincubation. The cell precipitate was resuspended with Annexin V‐binding buffer (BioLegend), 1 µL CD326 antibody, and 1 µL Annexin V antibody (BioLegend) were added into the cell suspension and incubated for 15 minutes at 4°C in the dark. CD326 was mainly marked the tumor epithelial cells. Propidium iodide (PI; Sigma) was added to the cell suspension before testing. The sum of Annexin5 single‐positive rate (early apoptosis) and PI, Annexin5 double‐positive rate (late apoptosis) were the apoptosis rate of tumor cells. All samples were analyzed using FACSCanto II or C6 flow cytometer (Becton Dickinson), and data were analyzed using FlowJo software (FlowJo, LLC, Ashland).

### 2.8 RNA extraction and qRT‐PCR

Total RNA was extracted from tumor and non‐neoplastic tissues by TRIzol reagent (Invitrogen), according to the operation protocol. Then, the RNA was reverse transcribed into cDNA using the PrimeScript RT reagent Kit with gDNA Eraser (TaKaRa). qRT‐PCR was performed using SYBR Premix Ex Taq II (TaKaRa) in Agilent Mx3005P. The following primers for MUC1 were, forward 5ʹ‐TTTCCAGCCCGGGATACCTA‐3ʹ and reverse 5ʹ ‐AGAGGCTGCTGCCACCATTA‐3ʹ. Glyceraldehyde 3‐phosphate hydrogenase (GAPDH) was used for normalization of data. The data were analyzed by 2^−△△Ct^.

### 2.9 Enzyme‐linked immunosorbent assay (ELISA)

The supernatant was collected after coculture of T and tumor cells. IL‐2, IFN‐γ, TNF‐α, and IL22 proteins, respectively, detected by human IL‐2, IFN‐γ, TNF‐α, and IL22 Quantikine ELISA kits (R&D Systems). Each protein should draw a standard curve and each sample had duplicated wells. The concentrations of the proteins were calculated by detecting the optical density (OD) value of absorbance at 450 nm. The results were calculated with the help of Excel (Microsoft).

### 2.10 TCGA database analysis

The expression of MUC1 of HNSCC was obtained from The Cancer Genome Atlas in 2016 (TCGA; https://cancergenome.nih.gov/). All data were analyzed using GraphPad Prism 7 software (GraphPad, Inc).

### 2.11 Xenograft animal model of head and neck squamous cell carcinoma

Animal experiments were approved by the Animal Ethics Committee of the First Affiliated Hospital of Zhengzhou University. All animal experiments were performed in accordance with animal laboratory care and experimental guidelines. Nonobese diabetic/severe combined immunodeficient (NOD/SCID) mice were purchased from Beijing Vital River Laboratory Animal Technology Co., Ltd. According to the Animal Ethics Committee, 6‐8‐week‐old female mice were bred and kept in a pathogen‐free environment.

Mice were randomly divided into four groups, respectively PBS, GFP T, CAR‐MUC1 T, and CAR‐MUC1‐IL22 T (5 mice/group). Every mouse was subcutaneously inoculated with 10^6^ HN4‐luc cells. After 10 days, 10^7^ GFP T cells, CAR‐MUC1 T cells, and CAR‐MUC1‐IL22 T cells were resuspended in 100 µL PBS and injected by tail vein injection, the PBS group was also injected 100 µL PBS. The maximum and minimum diameters of the tumor were measured with calipers, and tumor volume was calculated according to the formula *V* = (length × width^2^)/2. The mice were weighed with a weighing apparatus. Every 10 days, mice were anesthetized with 3% isoflurane (RWD Life Science) in an induction chamber. Anesthesia on the stereotactic frame (David Kopf Instruments) was maintained at 2% isoflurane delivered through a nose adaptor. 100 µL of D‐Luciferin was intraperitoneally injected sodium solution (0.15 mg/mL, Yeasen Biotech Co., Ltd.) with a blunt‐ended needle. After 10 minutes, Bioluminescence imaging was conducted on an IVIS Lumina, Series III spectrometer (Caliper Life Science) and was quantified using live image 4.3.1 software (PerkinElmer).

### 2.12 Statistical analysis

Data are presented as means *§* standard errors of the means. Student's *t* test, one‐way ANOVA were used to determine the statistical significance of differences between samples, and *P* value < .05 was considered to indicate a significant difference. All data were analyzed using GraphPad Prism 7 software (GraphPad, Inc).

## 3 RESULTS

### 3.1 MUC1 is commonly high expressed in HNSCC

**Figure 1:**
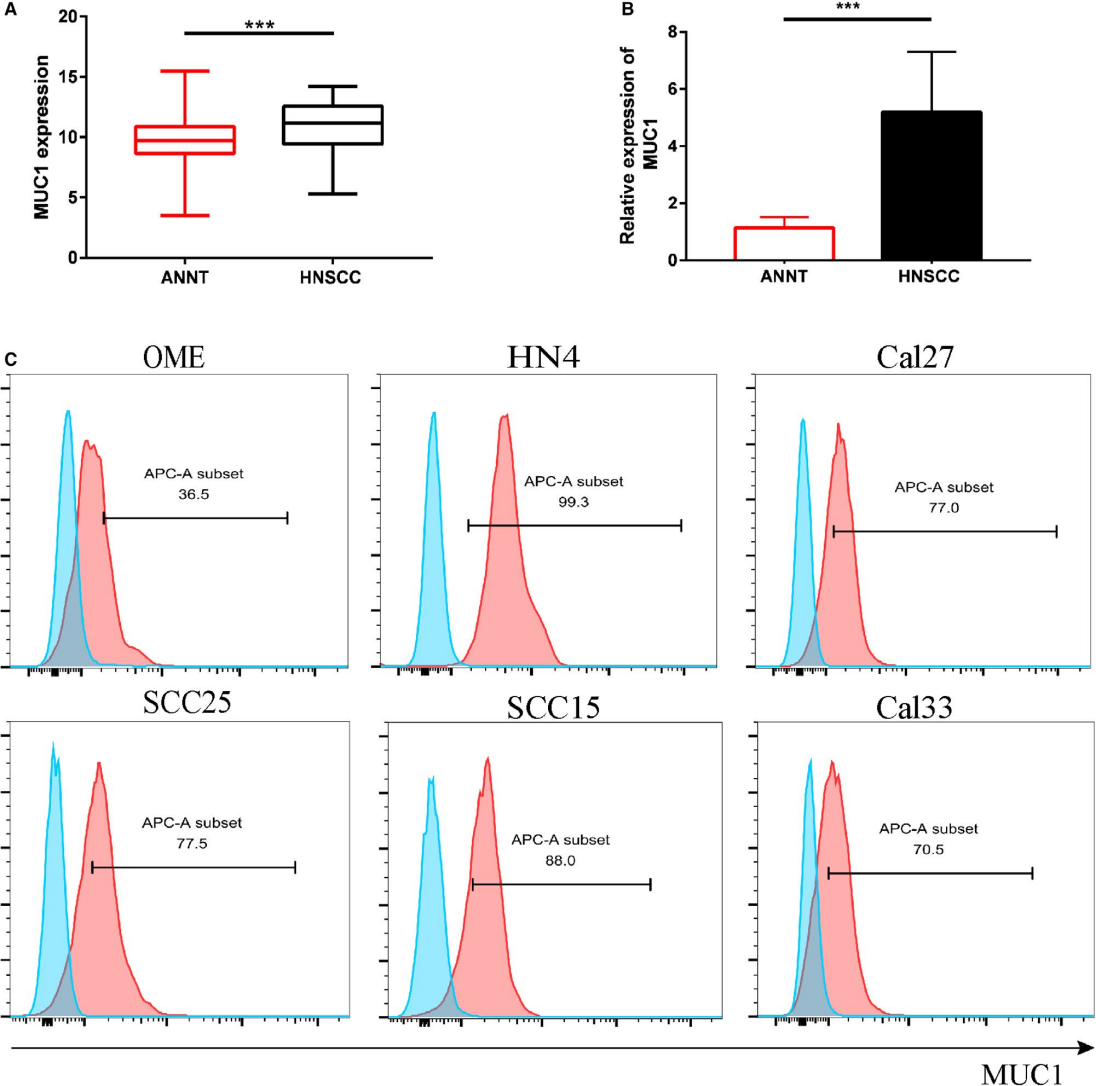
MUC1is commonly high expressed in HNSCC. A, The expression of MUC1 in HNSCC group vs normal group from TCGA database. Statistical significance was determined by unpaired t test. B, The gene levels of MUC1 were examined in 52 HNSCC tissues and adjacent non‐neoplastic tissues by qRT‐PCR. C, The MUC1 cell surface expression in six HNSCC cell lines by flow cytometry. Blue‐filled histograms represent control group without antibody; whereas the red‐filled histograms show staining with APC‐conjugated anti‐MUC1 mAb (monoclonal antibody). (Error bars represent the mean ± SEM. ***P < .001; ns, not significant)

To investigate the MUC1 expression in human HNSCC samples and cell lines, we exported data on the MUC1 gene in HNSCC (n = 2752) and ANNT (n = 521) from the TCGA database and performed statistical analysis. The result was shown in Figure 1A, MUC1 was the markedly high expression in human HNSCC at the mRNA level (*P* < .001). In addition, we founded that MUC1 expression was higher in 52 HNSCC tissues compared with ANNT by qRT‐PCR (Figure 1B). In human HNSCC cell lines (HN4, Cal27, Cal33, SCC15, SCC25), the MUC1 expression was higher compared with the OME epithelial cell line (Figure 1C). In view of positive MUC1 expression in HNSCC tissues and cell lines, MUC1 was a potential biomarker for the treatment in HNSCC.

### 3.2 Chimeric antigen receptor‐mucin 1(CAR‐MUC1) T cells specifically killed MUC1^+^ HNSCC cell lines in vitro

**Figure 2:**
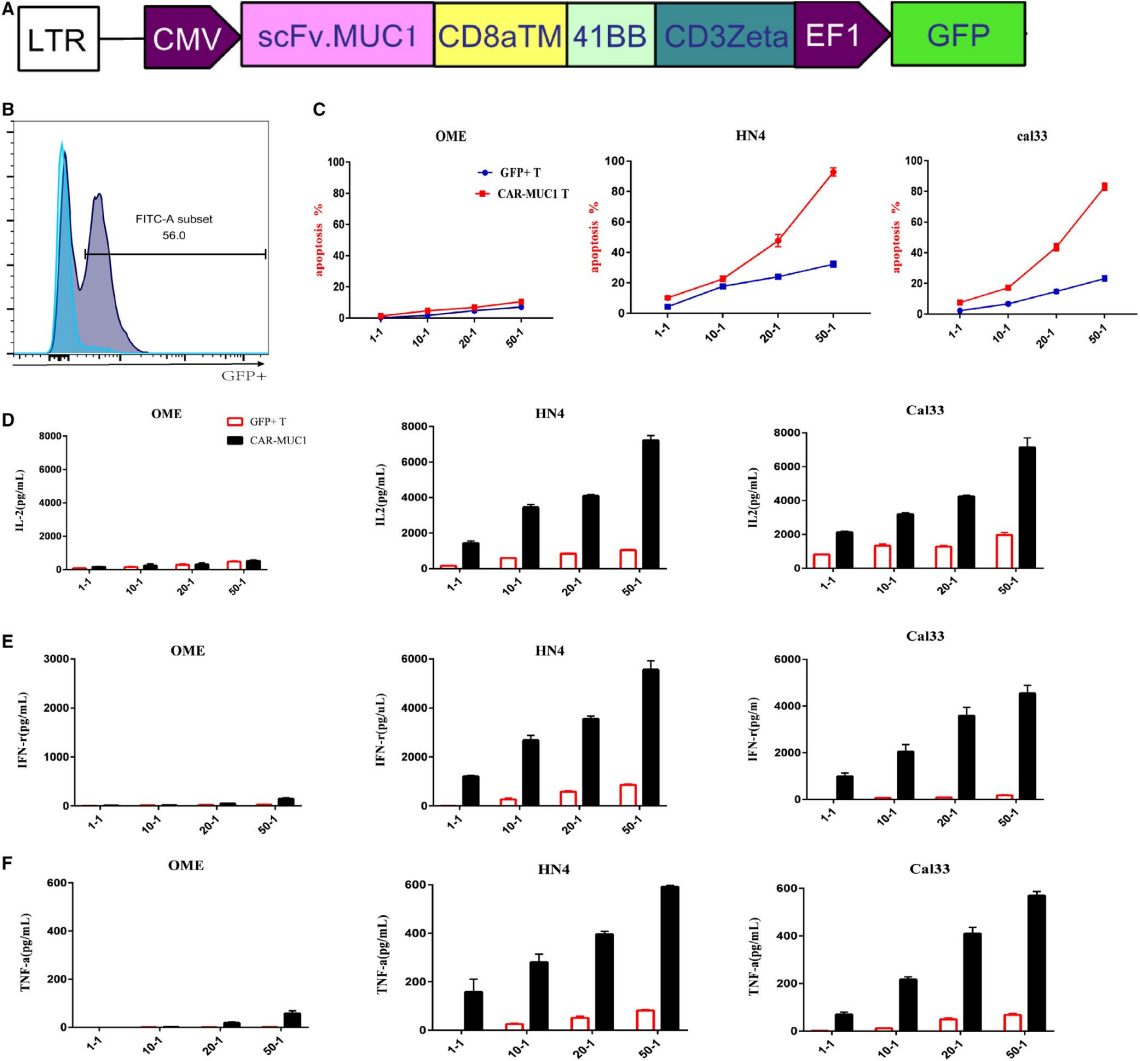
Chimeric antigen receptor‐mucin 1(CAR‐MUC1) T cells specifically killed MUC1^+^ HNSCC cell lines in vitro. A, Construction of CAR‐MUC1. B, Transfect efficiency of CAR‐T cells. Flow cytometry tested the positive rate of GFP compared with nontransfection. (The blue is control group and gray is positive group.) C, Flow cytometry tested the lysis of different tumor cells by CAR‐MUC1 T cells and GFP^+^ T cells, respectively. CAR‐MUC1 T and GFP^+^ T cells were coculture with tumors cell from OME, HN4, Cal33 for 6 h, respectively. The results were the sum of Annexin5 single‐positive rate (early apoptosis) and PI, Annexin5 double‐positive rate (late apoptosis). D‐F, IL‐2, IFN‐γ, and TNF‐α secretion of CAR‐MUC1 T cells and GFP^+^ T cells in coculture supernatants after different E/T ratio were measured by ELISA assay. Each trial was repeated three times, the T cells came from three different healthy donors. Both CAR‐T and GFP^+^ T cells in each trial came from the same volunteer

We evaluated the efficacy of CAR‐MUC1 T cells against HNSCC cell lines in vitro. First, we constructed a second‐generation CAR, which consisted of the scFv sequence derived from the anti‐MUC1 mAb (VH: HMFG2, VL: SM3),^20^ 4‐1BB signaling domains, the CD3ζ signal transduction area and followed by a green GFP^+^ signal. We packaged second‐generation CAR plasmids as lentiviral and transfected them into the sorted primary human CD3^+^ T cells. Transfection efficiency was measured by the fluorescence signal intensity of GFP^+^ (Figure 2B). Similarly, we transfected CD3^+^ T cells with null vector plasmid carrying GFP, and the GFP^+^ T cells as the control group.

We selected three cell lines, OME (an immortalized epithelial cells lines with low expression of MUC1 was used as the control group), HN4 (oropharyngeal carcinoma cell line), and Cal33 (tongue carcinoma cell line with high expression of MUC1 were used as the experimental groups). The cytotoxic function of GFP^+^ T cells and CAR‐T cells against tumor cells was observed at 6 hours coincubation with different E/T ratios (1:1, 10:1, 20:1, 50:1) (Figure 2C). Later, we detected the production of IL‐2, IFN‐γ, and TNF‐α functional cytokines in the supernatant of the cytotoxic assay (Figure 2D/E/F). These results indicated that compared with GFP + T cells, CAR‐T cells showed superior cytotoxic function against tumor cells, which was depended on the expression of MUC1.

### 3.3 IL22 induces upregulation of MUC1 expression in HNSCC and the changes of T‐cell function

The traditional second‐generation CAR‐T cells lead to complete apoptosis of cells when E/T was 50:1(Figure 2C). This led us to look for alternative methods in which we could achieve complete apoptosis of tumor cells at a lower E/T ratio.

**Figure 3:**
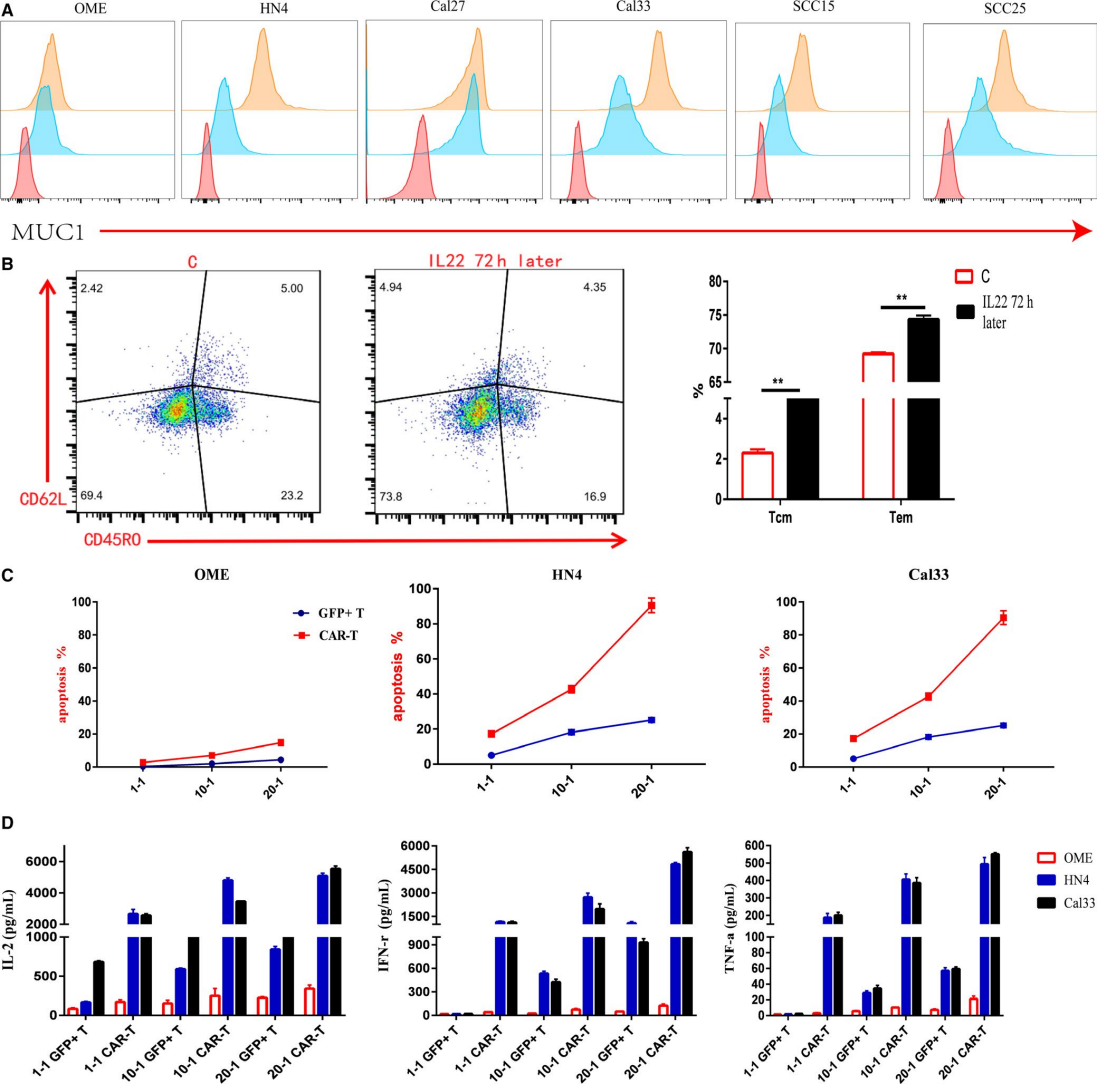
IL22 induces upregulation of MUC1 expression in HNSCC and the changes of T‐cell function. A, Exogenous addition of human IL22 recombinant protein could induce upregulation of MUC1 expression in HNSCC cell lines except OME and Cal27, which is highest at 72 h. The red is negative control; the blue is MUC1 expression; the orange is MUC1 expression after addition IL22 recombinant protein for 72 h. B, The left panel is normal CD3^+^ T‐cell differentiation; the middle panel is the characterization of CD3^+^ T‐cell differentiation by exogenous addition the IL22 recombinant protein for 72 h (Naïve: CD45RA^+^/CD62L^+^; Central Memory: CD45RA^−^/CD62L^+^; Effector Memory: CD45RA^−^/CD62L^−^; Effective: CD45RA^+^/CD62L^−^). The right panel is the statistical table. C, After 72 h of exogenous addition of IL22 recombinant protein, detected the killing of CAR‐MUC1 T cells against OME cell line and different HNSCC cell lines in different E/T ratios. The results were the sum of Annexin V single‐positive rate (early apoptosis) and PI, Annexin V double‐positive rate (late apoptosis). D, After 72 hours of exogenous addition of IL22 recombinant protein, cytokine secretion in coculture supernatants after different E/T ratio were measured by ELISA assay. (Error bars represent the mean ± SEM. **P < .01; ns, not significant)

In the previous study, IL22 was reported to induce upregulation of MUC1 expression in colon cancer due to the presence of inflammatory microenvironment.^21^ Upon that, we investigated whether IL22 (20 ng/mL) could upregulate MUC1 expression in HNSCC cell lines by flow cytometry. Although there was an increase at 24 and 48 hours, the MUC1 expression reached the highest at 72 hours except for the OME and Cal27 (Figure 3A). In view of the effect of IL22 on MUC1, we want to verify whether IL22 influences T cells function. Next, we detected the change of T cells function following the addition of IL22 recombinant protein for 72 hours. Figure 3B showed the CD3^+^ T‐cell differentiation results detected by flow cytometry (Naïve: CD45RA^+^/CD62L^+^; Central Memory: CD45RA^−^/CD62L^+^; Effector Memory: CD45RA^−^/CD62L^−^; Effector: CD45RA^+^/CD62L^−^). After 72 hours of exogenous addition of IL22 recombinant protein, we found that CD3^+^ T cells slowly differentiated into central memory and effective memory.

After 72 hours of exogenous addition of IL22 recombinant protein in HNSCC cell lines, the killing ability at different E/T ratios after 6 hours of GFP^+^ T cells and CAR‐MUC1 T cells were detected. We found that the tumor cells underwent apoptosis at the E/T ratio of 20:1. There was no significant change in apoptosis of OME cells (Figure 3C). Next, we detected the secretion of IL‐2, IFN‐γ, and TNF‐α in the supernatant of the cytotoxic assay (Figure 3D). In conclusion, exogenously added IL22 recombinant protein can enhance the cytotoxic ability of CAR‐MUC1 T cells depended on the increasing stronger expression of MUC1 and the changes in T‐cell differentiation.

### 3.4 CAR‐MUC1‐IL22 T cell show improved cytotoxic function

**Figure 4:**
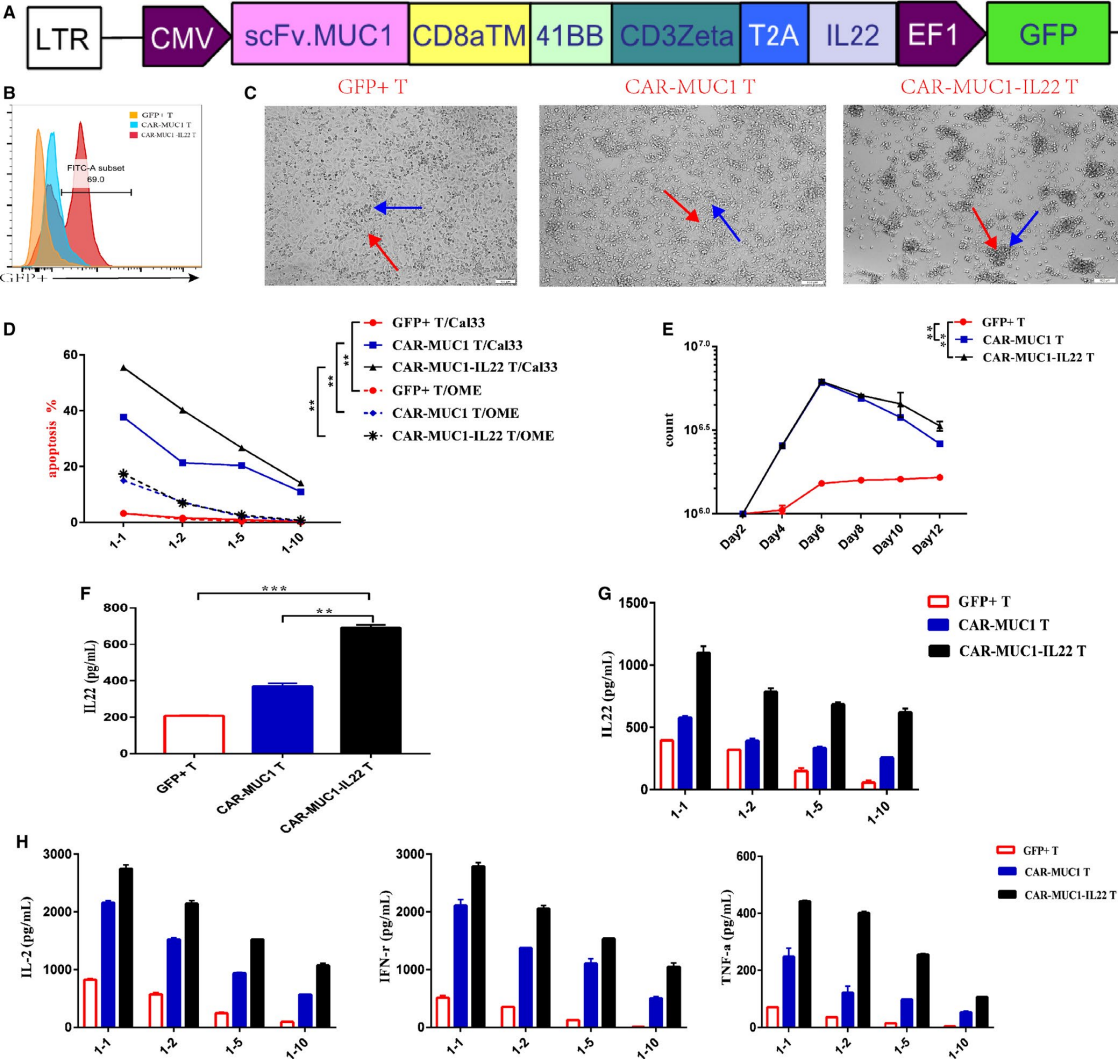
CAR‐MUC1‐IL22 T cell show improved cytotoxic function. A, Construction of CAR‐MUC1‐IL22. The IL22 CDS region is behind the CAR‐MUC1 structure by 2A peptide. B, T‐cell transfection efficiency of two kinds CAR‐T cells. Flow cytometry tested the positive rate of GFP in transfection when compared with nontransfection. (The orange one is control group, the blue one is the second generation CAR group and the red one is the forth‐generation CAR group). C, Microscopic observation of the ability of two different kinds of CAR‐T cells and GFP^+^ T cells to capture cells in 1:2 E/T ratio. In GFP^+^ T cells group, T cells are scattered around the tumor cells; In CAR‐MUC1 T cells group, a little number of CAR‐MUC1 T cells agglomerate around tumor cells; In CAR‐MUC1‐IL22 T cells group, large number of CAR‐MUC1‐IL22 T cells agglomerate around tumor cells (Red arrow is point to the tumor cell; the blue arrow is point to the T cell or two kinds of CAR‐T cells). D, Three different T cells were incubated with tumor cell (Cal33) and non‐neoplastic epithelial cell (OME) at a low E/T ratio (1:1; 1:2; 1:5; 1:10) for 72 h, flow cytometry tested the apoptosis rate of tumor cells (*P* < .01). The results were the sum of Annexin5 single‐positive rate (early apoptosis) and PI, Annexin5 double‐positive rate (late apoptosis). E, Representative T‐cell expansion profile of three different T cells (*P* < .01). F, ELISA detected the secretion of IL22 in the culture supernatant (*P* < .01). G, ELISA detected the secretion of IL22 in the coculture supernatant of different E/T ratio. H, ELISA detected the secretion of IL‐2/IFN‐γ/TNF‐α in the coculture supernatant of low E/T ratio. Each trial was repeated three times, the T cells came from three different healthy donors. Both CAR‐T cell and T cell in each trial came from the same volunteer. (Error bars represent the mean ± SEM. ***P < .001; **P < .01; ns, not significant)

To reduce the total amount of retransfusion T cells, increase antigen expression, and further improve CAR‐T‐cell function, we constructed a fourth generation CAR structure that could secrete IL22 (Figure 4A). We verified the killing capacity of the fourth generation CAR‐T in vitro. First, we detected the transfection efficiency of the two different kinds of CAR‐T cells (Figure 4B). To intuitively show the state changes of T cells coincubated with tumor cells, we took photos of the cells after plating, at an E/T ratio of 1:2, the time was 48 hours. We can see that the formation of CAR‐T cells clusters and the lysis of Cal33 tumor cells under microscope, CAR‐MUC1‐IL22 T cells have better proliferate than CAR‐MUC1 T cells; whereas the GFP+T cell did not proliferate and gradually died. (Figure 4C). The highest MUC1 expression was achieved 72 hours after IL22 cytokine addition, all the cytotoxic experiments were performed at the 72nd hour to detect the apoptosis of tumor cells. The E/T ratio used was 1:1, 1:2, 1:5, 1:10, respectively. Figure 4D shows the statistics graph of apoptosis in Cal33 cells and OME cells (*P* < .01).

Next, we detected the proliferation of three different T cells 48 hours after transfecting plasmid. Among them, GFP^+^ T cells proliferated slowly, and the other two CAR‐T cells proliferated faster. However, due to lentivirus transfection, the number of CAR‐T cells decreased gradually (*P* < .01) (Figure 4E). Reportedly, IL22 was mainly produced by CD4^+^ T cells and CD8^+^ T cells,^22^ so we tested the secretion of IL22 when T‐cell number was constant and on 48 hours after transfecting plasmid. (Figure 4F) After 48 hours, T cells will proliferate in different numbers. (Figure 4E) The more proliferation, the more IL22 secrete. Additionally, we used ELISA assay to detect the secretion of IL22 (Figure 4G) and the secretion of other functional cytokines in the supernatant of T cells coincubated with Cal33 for 72 hours, such as IL‐2, IFN‐γ, and TNF‐α (Figure 4H). We discovered that the smaller the E/T ratios, the weaker the cytotoxic function of T cells, the slower the proliferation of T cells, and the weaker the ability to secrete functional cytokines. Moreover, CAR‐T cells secreting IL22 have a stronger cytotoxic function.

### 3.5 CAR‐T cells induce tumor degradation of HNSCC in vivo

**Figure 5:**
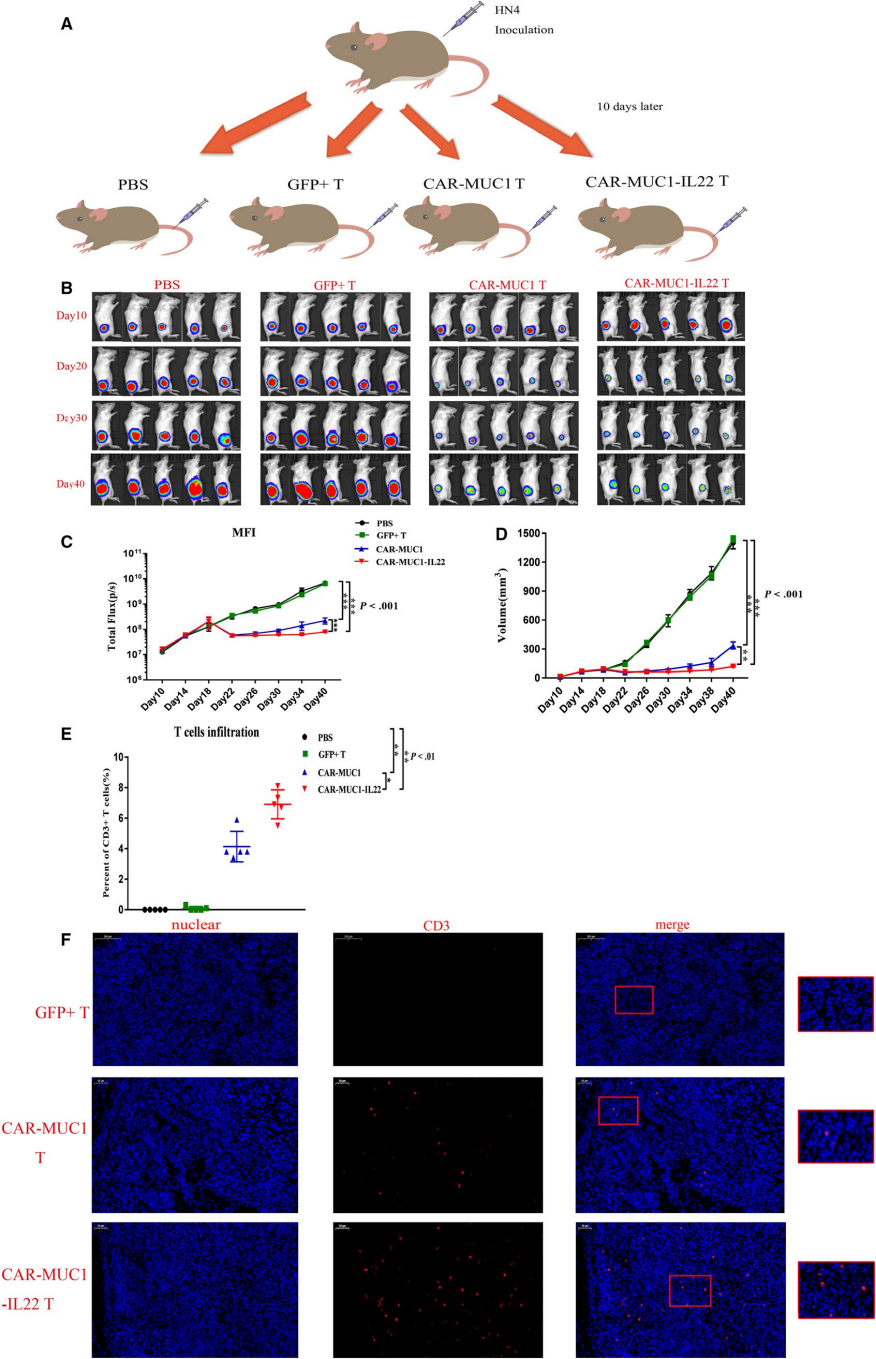
CAR‐T cells induce tumor degradation of HNSCC in vivo. A, Mouse model of HNSCC: 10^6^ *f*Luc^+^ HN4 tumor cells were injected into the subcutaneous tissue in NSG mouse. After 10 d, tail intravenous injection of 100 µL PBS (n = 5), GFP^+^ T cells (n = 5), CAR‐MUC1 T cells (n = 5), CAR‐MUC1‐IL22 T cells (n = 5). The mice were sacrificed on day 40 for tumor analysis. B, Bioluminescence images of four treatment groups mice. C, Total body bioluminescence units quantitate were measured by bioluminescence photometry and flux values (photons per second, *P* < .001). D, Tumor volume growth curves of four treatment groups (*P* < .001). E, T‐cell infiltration statistics in four treatment groups (*P* < .01). F, CD3 in tumor tissue was detected using the anti‐CD3 antibody (Abcam 5690, dilution 1:200); cell nuclei were stained with DAPI. Scale bar represents 200 µm. (Error bars represent the mean ± SEM. ***P < .001; **P < .01; *P < .05; ns, not significant)

We used the NOD/SCID mouse model to confirm the efficiency of CAR‐T cells. First, we randomly divided 20 immuno‐deficient mice into four groups, five mice per group. Each mouse was subcutaneously inoculated with 10^6^ *f*Luc^+^ HN4 cells. After 10 days, mice in each group were injected with 100 µL PBS, 100 µL 10^7^ GFP^+^ T cells, 100 µL 10^7^ CAR‐MUC1 T cells, or 100 µL 10^7^ CAR‐MUC1‐IL22 T cells by tail injection, respectively (Figure 5A). PBS‐treated mice were the control group. Bioluminescence photographs were taken every 10 days, and the mice were killed for analysis on the 40th day. Quantification of bioluminescent imaging showed exponential tumor growth in the control group, receiving PBS, and those receiving GFP^+^ T cells. Mice receiving CAR‐MUC1 T and CAR‐MUC1‐IL22 T cells showed a significant decrease in tumor growth. After tail vein transfusion, the tumor volume and fluorescence intensity continued to increase in PBS and GFP^+^ T group; however, the tumor grew slowly and remained stable in the two CAR‐T groups, after the CAR‐T cells transfusion. Moreover, tumor growth was slower in the CAR‐MUC1‐IL22 T cells group than in the CAR‐MUC1‐T cells group. (GFP^+^ T vs CAR‐MUC1 T, *P* < .001; GFP^+^ T vs CAR‐MUC1‐IL22 T, *P* < .001; Figure 5B‐C). The tumor volumes in HN4 xenograft mice treated with GFP^+^ T or CAR‐T were significantly different (*P* < .001; Figure 5D). Flow cytometry was used to test the tumor infiltration of CD3^+^ T cells in each group (Figure 5E). Immunofluorescence revealed the infiltration of CD3^+^ T lymphocytes in tumor tissues (Figure 5F). In the GFP^+^ T group, there was almost no infiltration of CD3^+^ lymphocytes; in the CAR‐MUC1 T group, the infiltration of CD3^+^ lymphocytes was moderate; in the CAR‐MUC1‐IL22 T group, the infiltration of CD3^+^ lymphocytes was the highest compared with other groups. Collectively, these results proved that the CAR‐MUC1‐IL22 T cells and CAR‐MUC1 T cells exhibited superior efficacy against HNSCC, compared with GFP^+^ T cells in vivo.

## 4 DISCUSSION

Studies have shown that the incorporation of costimulatory molecules could enhance the secretion of cytokines, the proliferation of CAR‐T cells and tumor regression.^23^ Most CARs related basic and clinical trials have tested on second‐generation CAR. In our research, we first verified the cytotoxic ability of the second‐generation CAR in HNSCC cell lines, but, the tumor cells should complete apoptosis when the E/T ratio reached 50:1. In order to reduce the total amount of transfusion T cells, we used the fourth‐generation CAR‐T cells that could secrete the IL22 cytokine and coincubated them with tumor cells.

The tumor cells underwent 60% apoptosis when the E/T ratio was 1:1 at 72th hours. It is mainly because IL22 could induce the expression of MUC1, allowing CAR‐T cells to bind more accurately to tumor cells. At the same time, IL22 also upregulate the proportion of central memory and effective memory T cells in CD3^+^ T cells, allowing T cells to proliferate and survive longer. Our in vivo experiments have also proved that, after transfusion of cells, the tumor volumes were obviously increased in control group. However, the tumor volumes were significantly decreased in two CAR‐T cells groups, and until the 20th day after transfusion, the tumor volumes begin to increase slowly, among that, the CAR‐MUC1‐IL22 T cells group increased most slowly. These were mainly due to the short survival time of the second‐generation CAR‐T cells in vivo, whereas IL22 can facilitate the differentiation of CAR‐T cells into a T_CM_ phenotype and improve the survival of the CAR‐T cells. To analyze the infiltration of CD3^+^ T cells, the mice were sacrificed on the 40th day. The survival of the mice and CAR‐T cells homing function, when reseeded with tumor cells could be improved by future studies.

Exogenous addition of certain cytokines could enhance the function of T cells. For instance, Keishi et al designed fourth‐generation CAR‐T cells that secrete IL‐7 and CCL19, among which IL‐7 can enhance the proliferation and survival of T cells.^24^ CCL19 is a chemokine that can recruit T cells and DC cells.^25^ Yang et al combined the use of IL‐7 and IL‐15 to maintain the T‐memory stem cells phenotype and enhance CAR‐T function.^26^ Despite these findings, the intricate mechanisms by which cytokines enhance T‐cell efficacy remains difficult to apply to the clinic and the results are not very satisfactory.

The high efficiency of T cells transfection from CAR is another reason. The transfection efficiency is too low, and the CAR‐T‐cell function is weaker. Moreover, the transfection efficiency is also related to the donor. Different PBMC donors have different CD4^+^/CD8^+^ T‐cell ratios. Transfected T cells due to the addition of viral plasmids, the proliferation rate of T cells slows down and final apoptosis. According to the current technologies available in our laboratory, the survival time of T cells after GFP+ sorting is short. Prolonging the survival time of T cells is also the focus of our work in the later stage. Therefore, ensuring high transfection efficiency and T cells longer survival time can make CAR‐T cells have a strong killing function.

The weak ability of tumor antigen recognition is the main reason for the poor effect of CAR‐T treatment. Enhancing the specificity of antigen can not only increase the efficacy but also avoid the off‐target effect. CAR‐T therapy is so effective in treating hematomas malignancies, mainly diffuse large B‐cell lymphoma and B‐cell acute lymphoblastic leukemia, because CD19 and CD20 are relatively tumor‐specific antigens (TSAs) expressed in many of these cancers, making the targeted antigens CAR‐T more effective. One of the reasons why CAR‐T therapy is not ideal in treating solid tumors is because solid tumors express more tumor‐associated antigens (TAAs) and less TSAs. TAAs are present on the surface of tumor cells and normal cells, and are highly expressed when tumor cells proliferate, and normal cells can also be expressed in a small amount. When using CAR‐T to treat solid tumors, it often causes off‐target effects due to simultaneous attack on normal cells and tumor cells, resulting in unsatisfactory results. For example, human epidermal growth receptor factor 2 (Her2) is an important prognostic factor for breast cancer; however, Her2 is also highly expressed in other solid tumors, such as pancreatic ductal adenocarcinoma, glioblastoma, and is not completely specific like CD19 and CD20. Most solid tumors express a variety of TAA instead of TSA.

Although numerous TAAs have been detected in HNSCC, the current literature on CAR‐T treatments report those targeting CD70^27^ and human epidermal growth receptor factor 2 (Her2).^28^ Therefore, it is important to broaden the HNSCC‐specific targets of CAR‐T cells. Once a specific antigen has been identified, CAR‐T treatment can reduce the “off‐target” effect and kill the target cells accurately.

Our rationale for developing a MUC1 targeted therapy for the treatment HNSCC is based upon the biological properties of MUC1.^29^ MUC1 has been reported in HNSCC^30^ and breast carcinomas which are associated with metastasis and invasion.^31^ The subunit of MUC1, MUC1‐C (MUC1 C‐terminal), was associated with TRAIL, Fas ligand, and inhibiting Perforin/ Granzyme B‐mediated lysis, thus supporting the idea that MUC1 contributes to immune evasion.^32^ MUC1‐C has also been reported to induce the PD‐L1 expression in breast cancer,^33^ increase stem cell characteristics in colon cancer^34^ and augment resistance to genotoxic anticancer agents.^35^ CAR‐T therapies have been reportedly used to target MUC1‐expressing cancers, such as non‐small‐cell lung carcinoma,^12^ pancreatic adenocarcinoma.^36^ In addition, clinical trials have been used targeting Tn glycoform of MUC1 in leukemia and pancreatic adenocarcinoma.^37^ However, it has not been used in HNSCC. Given the biological characteristics of MUC1, we have tried to explore new ways to treat HNSCC using MUC1.

At present, NOD/SCID mice lack a host immune system and do not produce immune rejection. CAR‐T cells can survive in vivo for a long time and are more suitable for cytokine transformation of CAR‐T tumor killing ability. However, the cascade reaction caused by cytokine release syndrome (CRS) in the human body cannot be completely simulated, and the interaction between CAR‐T cells and other facial cells or tissues in the body cannot be observed, and the off‐target effect cannot be detected. Therefore, our next step is to construct an immune system to reconstruct patient‐derived xenograft (PDX) mouse models of human HNSCC that maintained the antigenic profiles of primary tumors as well as laboratory studies of primary tumor cells. Growth of tumors in the human immune system can be studied, and the antitumor efficacy and related toxic mechanisms of CAR‐T cells can be evaluated. These mouse models are well tolerated by humanized CAR‐T cells and compensates for defects in homologous mouse models and transplanted mouse models. Thus models provide CAR‐T with more valuable effectiveness and safety information of HNSCC therapy.

## 5 CONCLUSION

In conclusion, CAR‐T‐cell modifications and the emergence of new antigens and technologies, are intended to promote the development of CAR‐T‐cell therapy and broaden the therapeutic experience on cancer from hematological malignancies to solid tumors. Our designed fourth‐generation humanized MUC1 CAR‐T cells provide excellent preclinical studies for patients with HNSCC with MUC1 expression. These findings could design safer and more effective CAR‐T cells to bring the best treatment to the patients with HNSCC in the future.

## CONFLICT OF INTEREST

None declared.
