# Correction to ‘MUC1 as a Target for CAR‐T Therapy in Head and Neck Squamous Cell Carcinoma’

**DOI:** 10.1002/cam4.70558

**Published:** 2024-12-30

Mei Z , Zhang K , Lam AK , Huang J , Qiu F , Qiao B , Zhang Y . MUC1 as a Target for CAR‐T Therapy in Head and Neck Squamous Cell Carinoma. Cancer Medicine 2020 Jan;9(2):640–652. 10.1002/cam4.2733 31800160 PMC6970025

Some incorrect images were used in Figure 5B. The correct figure is shown below. The authors confirm all results and conclusions of this article remain unchanged.

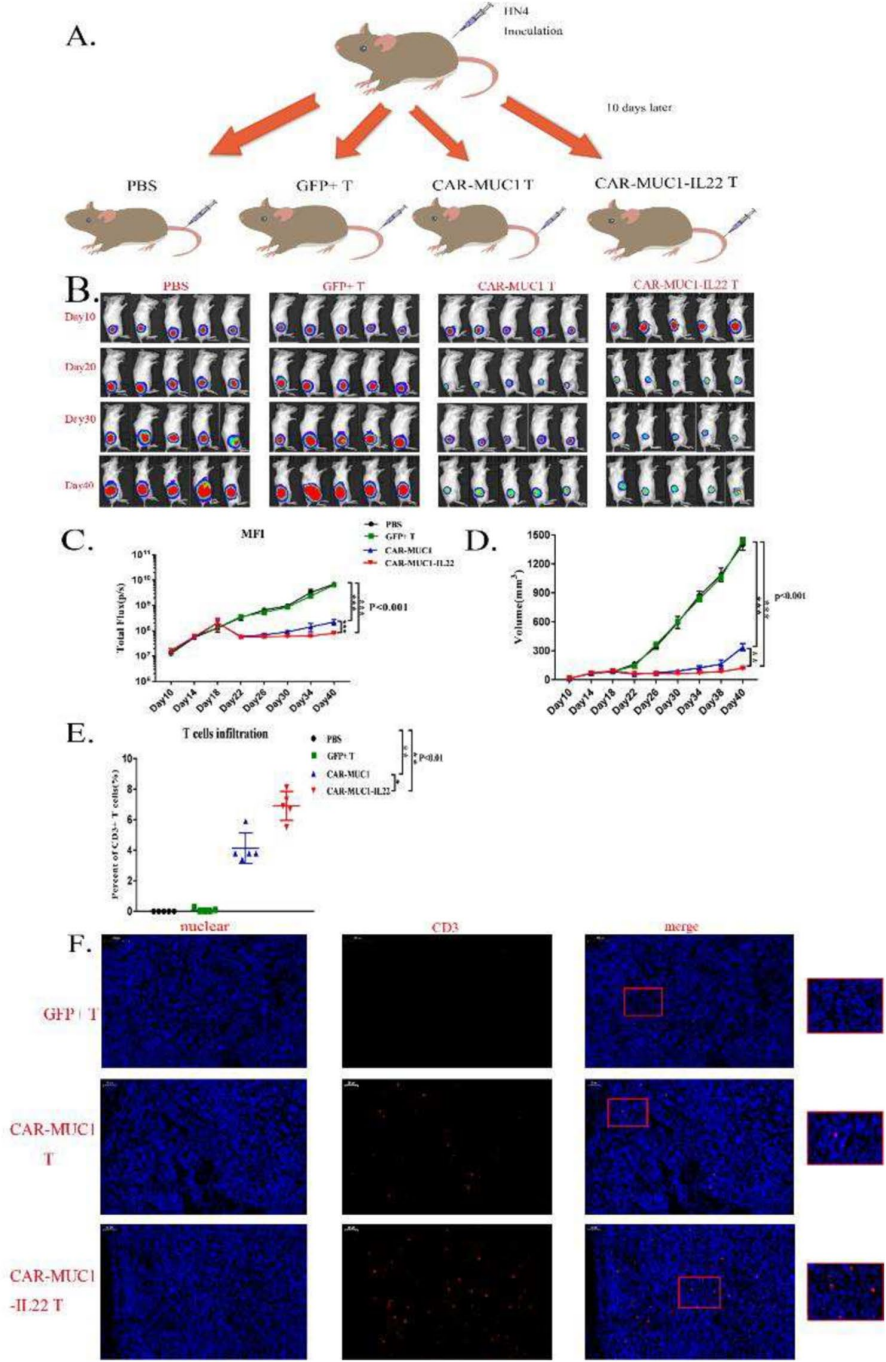

We apologize for this error.

